# Traumatic Neurasthenia

**Published:** 1916-03-04

**Authors:** 


### TRAUMATIC NEURASTHENIA.

### The Borderline of Malingering.

As a result of the Employers' Liability Act and of the Workmen's Compensation Act traumatic neurasthenia has become a plague and a nuisance to many members both of the legal and of the medical professions. Many definitions of the condition have been presented, and many tests for its detection formulated, yet it remains a fertile cause of disputes, and appears sometimes to lead to a good deal of hard swearing. Some of the lawyers have come to believe that it is a medical phantasy, born of the desire to help patients who are claiming damages for personal injury, and this creed would appear to have found its expression in the judicial utterance that traumatic neurasthenia is a disease invented for the purpose of securing damages from the London County Council. On the other hand, there is a strong body of medical evidence in favour of the reality of the condition, and no physician having experience of nervous diseases will fail to maintain this. The discredit attached to the term arises from two causes. First the word has been often used in quite inappropriate circumstances, and secondly, the Law Courts have learned by experience that what a claimant calls traumatic neurasthenia an insurance company terms malingering. From these influences prejudice is likely to arise against a plaintiff who bases his claim on the evidence of traumatic neurasthenia.

Another complexity of the situation, at least in its legal relations, is the difficulty of conveying to anyone who is destitute of a medical training the possibility of the existence of a condition which is neither organic disease on the one hand nor pretence adopted for the purposes of fraud on the other. If, argues the lay mind, the man has no physical damage and yet his arm hangs helplessly at his side he must be shamming. The temporary failure of function attendant upon shock, that is immediately following the application of violence, is realised easily enough; but not a failure which endures long after signs of general weakness and collapse have passed away. Indeed, were it not for actual clinical experience, realisation of such a condition would be anything but easy for the medical practitioner. But when by direct observation it is found that without any sign of physical injury there may be not only motor paralysis, but also such complete loss of sensation that the patient does not betray the slightest emotion when needles are thrust into his skin or a powerful electric current is applied to the surface, and that this condition may completely disappear, it becomes evident that the nerve centres associated with sensation may have their function temporarily suspended quite apart from damage to their physical integrity. And if centres related to sensation, why not those related to motion? Everyone recognises that under the influence of a blow, or under the stress of emotion, brain function may be suspended to such an extent that the victim loses consciousness and lies powerless and paralysed. Let it be suggested that in similar fashion a limited part of the brain may have its function suspended, not for a brief period but for weeks or months, and we get a foundation on which a conception of neurasthenia may be framed.

There are, however, refinements which sometimes puzzle even the elect. When there is a well-defined local paralysis or a quite conspicuous anæsthesia, there are medical methods which make a demonstration of the true nature of the case a comparatively simple matter. But when the motor and sensory functions are normal and the defect alleged is rather general than local, the position is not such a confident one. The claimant who maintains that since his accident he has been unable to concentrate his attention on his work or business cannot be analysed and scrutinised in the objective fashion which is possible when the symptom is, say, a paralysis of the limbs on one side of the body; and much the same is true of a man who presents "general weakness" as the cause of his disability. Indeed, in some cases of this order there are no confident medical criteria by which can be distinguished with certainty true functional disease from shamming or pretence. Put such complaints as headache or pain in the back instead of "general weakness" and the perplexity here presented still remains. It is in this region that acute differences arise in the judgment of different medical observers. All may allow that there is no evidence of organic disease, but while one will say the functional defect is real and means neurasthenia, another will say that it is simulated and means malingering. Perhaps in each instance the observer has examined the claimant at a single interview only, and his opinion rests rather on general than on medical grounds strictly so called. These general grounds may be quite sufficient to show that the man is untruthful, and that he pretends to some defects which are certainly assumed. Even then it is not quite certain that underneath the assumed disability there is not something substantial and real. Yet in a practical world it will be accepted that if a man is a liar in one direction his word ought not to be taken in others, and no one will pity him if he gets something less than justice. It is to establish such a position as this that dexterity is practised by the professional advisers of insurance companies and of other corporations against whom workmen's claims are not infrequent. A kind of medical detective police skill is cultivated, and traps are laid to entangle the sinner. Under modern conditions this has become a necessary branch of practice, and though it can hardly be numbered among the scientific achievements of the profession it no doubt helps to defeat fraudulent claims. The danger to the practitioner in such work is that his mind acquires a bias against any claimant who is free from evidence of all organic injury or disease. Still the work is of much interest, and, as already stated, it has its utility in the world of affairs.

